# High-Quality Green-Emitting Nanodiamonds Fabricated by HPHT Sintering of Polycrystalline Shockwave Diamonds

**DOI:** 10.1186/s11671-020-03433-7

**Published:** 2020-11-09

**Authors:** Vladimir Yu. Osipov, Fedor M. Shakhov, Kirill V. Bogdanov, Kazuyuki Takai, Takuya Hayashi, François Treussart, Anna Baldycheva, Benjamin T. Hogan, Christian Jentgens

**Affiliations:** 1 Ioffe Institute, Polytechnicheskaya 26, St. Petersburg, Russia 194021; 2 ITMO University, Kronverksky 47, St. Petersburg, Russia 197101; 3 Department of Chemical Science and Technology, Hosei University, 3-7-2, Kajino, Koganei, Tokyo, 184-8584 Japan; 4 Faculty of Engineering, Shinshu University, 4-17-1 Wakasato, Nagano, 380-8553 Japan; 5 Université Paris-Saclay, CNRS, ENS Paris-Saclay, CentraleSupélec, LuMIn, 91190 Gif-sur-Yvette, France; 6 College of Engineering Mathematics and Physical Sciences, University of Exeter, Exeter, EX4 4QF UK; 7 Optoelectronics and Measurement Techniques Research Unit, University of Oulu, 90570 Oulu, Finland; 8 Microdiamant AG, Kreuzlingerstrasse 1, 8574 Lengwil, Switzerland

## Abstract

We demonstrate a high-pressure, high-temperature sintering technique to form nitrogen-vacancy-nitrogen centres in nanodiamonds. Polycrystalline diamond nanoparticle precursors, with mean size of 25 nm, are produced by the shock wave from an explosion. These nanoparticles are sintered in the presence of ethanol, at a pressure of 7 GPa and temperature of 1300 °C, to produce substantially larger (3–4 times) diamond crystallites. The recorded spectral properties demonstrate the improved crystalline quality. The types of defects present are also observed to change; the characteristic spectral features of nitrogen-vacancy and silicon-vacancy centres present for the precursor material disappear. Two new characteristic features appear: (1) paramagnetic substitutional nitrogen (P1 centres with spin ½) with an electron paramagnetic resonance characteristic triplet hyperfine structure due to the *I* = 1 magnetic moment of the nitrogen nuclear spin and (2) the green spectral photoluminescence signature of the nitrogen-vacancy-nitrogen centres. This production method is a strong alternative to conventional high-energy particle beam irradiation. It can be used to easily produce purely green fluorescing nanodiamonds with advantageous properties for optical biolabelling applications.

## Introduction

Nitrogen impurities and vacancies are the predominant defects in the majority of natural and synthetic diamonds. Individual defects can collectively form defect complexes with up to 6 subunits [1, 2]. Of these defect complexes, nitrogen-vacancy (NV^−^) and, to a lesser extent, nitrogen-vacancy-nitrogen (NVN) centres have attracted significant interest, due to their non-blinking red and green photoluminescence, respectively [3, 4]. NV^−^ and NVN can be controllably generated in nanodiamonds. Nanodiamonds are widely recognised as non-toxic nanoparticles and hence can be used as long-term traceable labels in biomedical applications [5]. Nanocrystals with NV^−^ colour centres are also used for quantum sensing [3].

High-pressure, high-temperature (HPHT) synthesis of diamonds with conventional transition metal solvent catalysts is a standard industrial technique. It is exploited in many laboratories to grow diamond crystals with advanced lattice parameters. However, in recent years various non-conventional metal catalysts have begun to be widely used [6]. Such a method allows the use of nitrogen-containing organic additives and metal getters to controllably dope diamond across a range from high nitrogen content (up to ~ 1000 ppm), down to a much lower level (~ 50 ppm) [7, 8]. HPHT is also used to anneal diamond crystals and improve their crystalline quality, to decolour them, and to sinter nanocrystals into larger polycrystals.

The grouping of individual nitrogen defects into larger complexes in diamond under the influence of temperature (and pressure) has been extensively studied. Such complexes can serve as specific markers, recording the temperature history of completed crystallisation processes [1]. NVN and NV^−^ centres can be created by irradiating pristine nitrogen-containing diamonds with high-energy (2–14 meV) electrons or protons, or ~ 40 keV heavy ions. The irradiation creates vacancies in the diamond lattice. Subsequent annealing of the samples at temperatures in the 500–2000 °C range causes grouping of the defects [3, 9, 10]. Currently, the mass production of NV^–^ or NVN-containing nanodiamonds is typically realised by high-energy electron irradiation. However, this process is both expensive and time-consuming and requires specific sample preparation. Thus, it is worth developing alternative methods for colour centre creation in nanodiamonds, which do not require particle irradiation.

A new method was recently introduced to fabricate submicron-size diamond crystals, relying on sintering 5-nm detonation nanodiamonds (DNDs) in the presence of C–H–O liquid additives [11, 12]. Under HPHT sintering conditions, the added C–H–O organics behave as a supercritical fluid that induces fast diamond recrystallisation. Rapid recrystallisation is accompanied by a higher density of vacancies [13]. This method, however, suppresses the pristine colour centres present in the precursor 5-nm DNDs [14]. Hence, an alternative approach that can preserve these defects or even create other types during the HPHT sintering process is desirable.

**Fig. 1:**
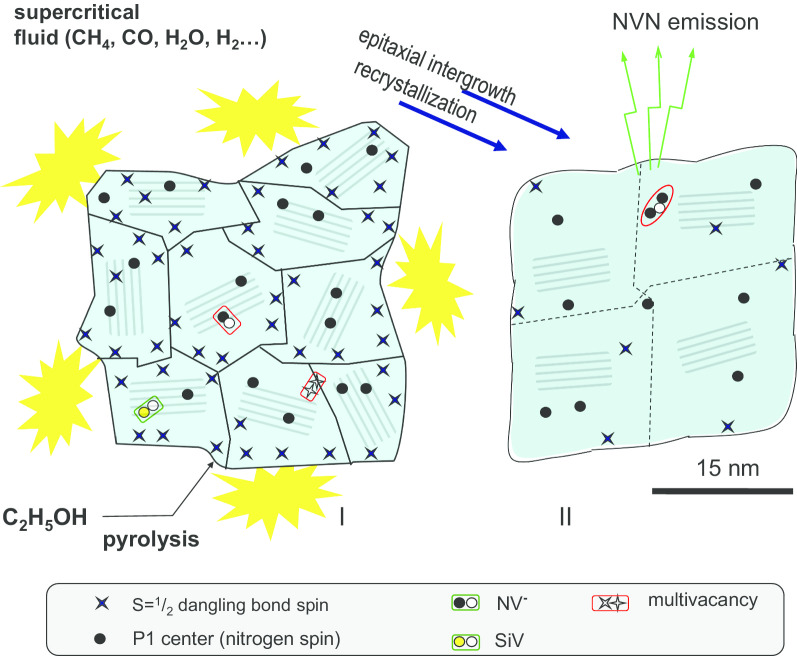
Schematic illustration of the sintering of polycrystalline diamond particles at high-pressure and high-temperature conditions to produce submicron diamond nanocrystals with sizes of about 30 nm. Various defects and colour centres are observed both in polycrystals (I) and in nanocrystals after the HPHT sintering (II)

In this work, we use precursor polycrystalline nanodiamonds produced by an explosion-assisted method using so-called DuPont shock-wave synthesis technology [15–17]. We sinter these nanodiamonds in the presence of C–H–O additives in the same way as in an existing patented procedure [18] (Fig. 1), obtaining coarse submicron diamonds containing only NVN colour centres, which were not present in the precursor nanoparticles. We also track the evolution of all NV^−^, P1, and SiV centres in the particles before and after the sintering, using electron paramagnetic resonance (EPR) and fluorescence to demonstrate the effect of the sintering on the full range of defects present.

## Methods

### Sample Fabrication

**Fig. 2:**
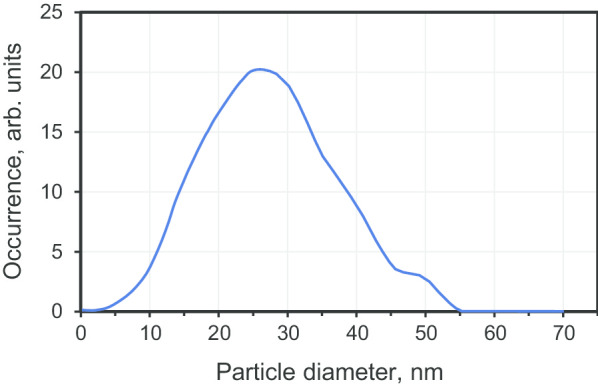
The size distribution of the DP 0–0.05 polycrystalline diamond fraction with a mean size ~ 27 nm measured by a differential centrifugal sedimentation apparatus (CPS disc centrifuge, CPS Instruments Inc., USA)

Submicron diamond crystals were prepared by sintering commercially available precursor polycrystalline nanodiamonds with a mean size of 25 nm (product DP 0–0.05, Microdiamant, Switzerland). The precursor particles are obtained by an explosive-assisted technique where a quick, shockwave-induced, graphite-to-diamond conversion occurs (within a hundred microseconds at *T* ≈ 950 °C, *P* ≈ 50 GPa). The products resulting from the explosion are subsequently chemically treated to purify the diamond nanoparticles phase, removing residual metals and amorphous carbon. The powder was composed of large polycrystals with sizes > 10 mμ. These polycrystals can be easily fractionated, by milling and disaggregation along grain boundaries, following a previously described method [19]. Size fractionation was undertaken to extract the small size fraction forming the DP 0–0.05 polycrystals. The resultant size distribution (Fig. 2) for the smallest fraction has its maximum at 27 nm and a full width half maximum (FWHM) ≈ 25 nm (with 95% of the particles with sizes in the range of 3–50 nm). The selected precursor particles are then transferred to the inner graphite cylinder of a toroidal-type high-pressure chamber for sintering. The typical sizes of the inner part of the high-pressure chamber (i.e. the above-mentioned hollow graphitic cylinder with two graphitic caps) were: inner diameter 4.0 mm and height 5.5 mm. Before sintering, ethanol was added dropwise to the dry nanodiamond powder until fully filling the interparticle space (around 30–50 wt%) [18]. Sintering then took place under high-pressure (7 GPa) and high-temperature conditions (1300 ± 50 °C) for 10 s. During one-press run, about ~ 120 mg of precursor diamond powder could be treated. The scheme of high-pressure chamber can be found in Ref. [11].

The effect of this sintering process is illustrated in Fig. 1. When sintering under HPHT conditions, the ethanol is in a supercritical state. Therefore, it can easily penetrate the polycrystalline grain boundaries, promoting diamond recrystallisation and new diamond phase growth. After sintering, ~ 90–100 mg of a white diamond powder of acceptable quality (without contamination from the graphitic container) was extracted from the high-pressure chamber (labelled D19 herein). The change of powder colour, from the grey/black of the precursor, to white after sintering indicates a change in the surface of the polycrystals and their corresponding growth [11].

### Structural Characterisation of the Materials Before and After the Sintering

#### Structural Characterisation of the Polycrystalline Nanodiamond Precursor

**Fig. 3:**
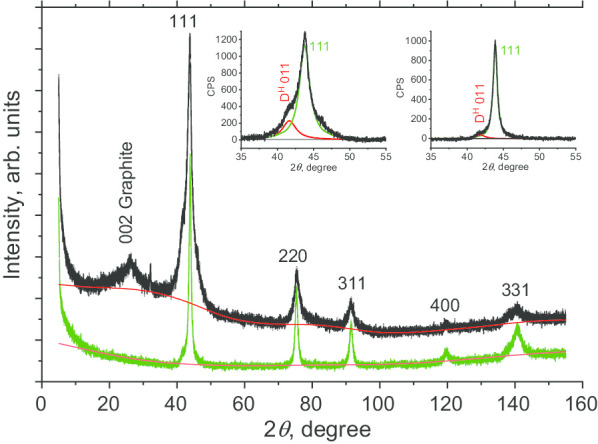
Powder XRD profiles of the DP 0–0.05 fraction of polycrystalline diamond particles (black curve) and D19 nanodiamonds obtained by sintering (green curve). Insets: Fitting of the most intense reflection from planes (111) with two (red and green) Lorentzian curves centred at 41.5° (D^H^ 011) and 43.8^o^ (D^c^ 111) for samples before (left inset) and after sintering (right inset)

We first characterised the structure of DP 0–0.05 before sintering. X-ray diffraction (XRD) measurements were taken with a Rigaku Smart Lab III X-ray Diffractometer, using CuKα radiation (*λ*= 1.54178 Å). A 40 kV voltage, 30 mA current, and a scan speed of 0.1 degree/min were used. The XRD pattern of DP 0–0.05 is shown in Fig. 3 (black) for 2*θ* in the range from 6° to 155°. We observe six peaks, five of which (111, 220, 311, 400, and 331) are characteristic of the diamond phase of carbon. The strongest observed peak is the cubic diamond (D^c^) 111 at 2*θ* ≈ 43.7°. This peak is not symmetrical, presenting a left shoulder. Fitting the pattern with two Lorentzians yields a first peak centred at 2*θ* = 43.76° and a second at 2*θ* = 41.64°. The first peak corresponds to reflection from (111) diamond planes, while the second (labelled D^H^ 011) is tentatively attributed either to: (a) reflections from planes of a hexagonal 6H polytype diamond phase [20] or to (b) multiple stacking faults, twins, and related grain boundaries between the crystallites under high stress. By comparing the integrals of the D^H^ 011 and D^c^ 111 peaks, we estimate the unidentified “phase” (either hexagonal or composed of structural defects) in the DP 0–0.05 precursor to represent ≈ 20 wt%. The peak labelled 002 Graphite (at 2*θ* ≈ 26°) is attributable to a nanographite phase. While this impurity phase can be easily removed, we intentionally did not etch with acid in this study. By comparing the areas of the 002 and 111 peaks, this amorphous phase was evaluated to contribute ~ 4 wt%. We then evaluated the coherent scattering region length (*L*_CSR_) using the Scherer method. We considered the FWHM *β* of the five diamond peaks as a function of sec *θ*. *Β* is determined after deconvolution from the diffractometer response function.^1^ The *L*_CSR_ for the DP 0–0.05 sample was found to be 6.7 nm.

**Fig. 4:**
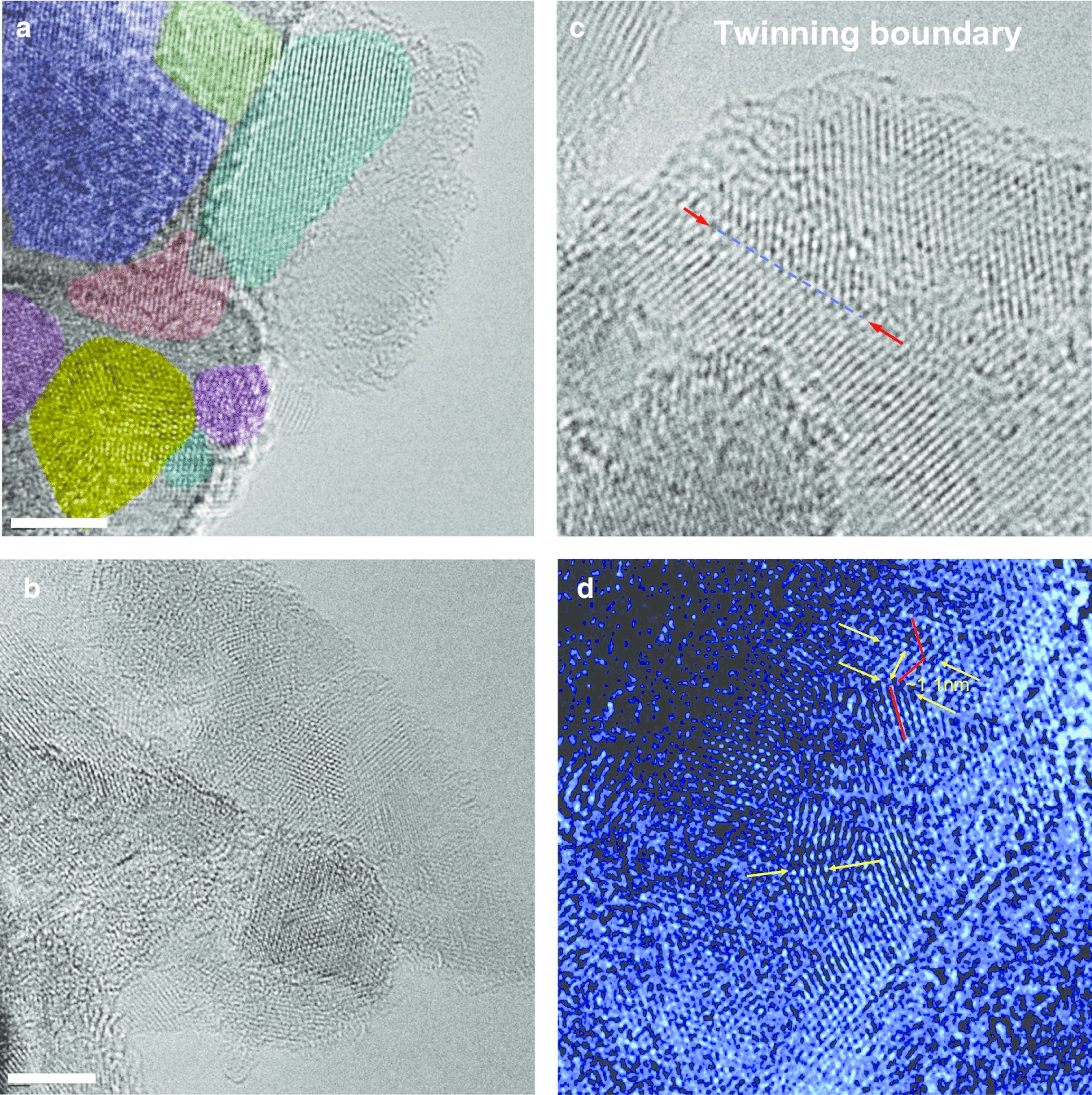
High-resolution TEM images of selected cubic diamond polycrystals extracted from the DP 0–0.05 powder (**a**, **b**) and typical images of the simple twinning boundaries, of several nanometres in length, found occasionally in the sample (**c**,** d**). Panels (**a**,** b**): scale bar—4 nm. Different crystallites are highlighted in different colours in (**a**). Arrows in panels (**c**) and (**d**) mark the selected clearly distinguishable twinning boundaries

To gain further insight into the complicated precursor DP 0–0.05 diamond polycrystal structure, we used a high-resolution transmission electron microscope (HRTEM, JEOL JEM-2100F equipped with CEOS Cs correctors, Shinshu University). The HRTEM was operated at 80 kV to minimise irradiation damage. The HRTEM images (Fig. 4) show that the polycrystals are composed of tightly bound cubic nanodiamond crystallites with characteristic 1.93 Å *d*-spacing fringes. The polycrystal imaged in Fig. 4a is composed of crystallites with sizes from 5 to 12 nm, having different orientations marked by different colours. Furthermore, HRTEM demonstrates that the size of the individual polycrystals composing the DP 0–0.05 powder is broadly distributed, in the 5–50 nm range. HRTEM images did not reveal any trace of a hexagonal diamond nanophase (like lonsdaleite). However, we observe many short twinning boundaries (Fig. 4c, d), some of them exhibiting pleated lattice planes with accordion shapes as in Fig. 4d. These observations indicate that the 111 XRD peak shoulder (Fig. 3) discussed previously is most likely due to stacking faults associated with multiple twinning-related grain boundaries rather than to the presence of a hexagonal diamond phase [16].

The elemental composition of DP 0–0.05 was analysed by the Pregl–Dumas method using an organic elemental analysis system (JM10, J-Science Lab Co., Ltd, Kyoto, Japan) in which the sample was burned at 1007 °C under an oxygen flow (30 ml/min). The oxygen fraction was determined by a balance. The results of the analysis, in wt%, are: C—90.45, N—2.47, H—0.76, O—6.32. The relatively large concentration of nitrogen means that almost all nitrogen is present inside the polycrystals in the aggregated form, probably in the form of A-centres (NN-dimers). Further analysis by X-ray fluorescence showed the presence of other trace elements in DP 0–0.05: Fe (~ 300 ppm), Cu (35 ppm), Si (120 ppm), Cr (~ 150 ppm), Ca (~ 45 ppm), Mn (45 ppm), P (30 ppm), Al (18 ppm), Ti (13 ppm), Mg (6 ppm), Ni (2 ppm), Zn (2 ppm), Co (1 ppm).

The DP 0–0.05 sample used for subsequent magnetic resonance studies was additionally treated in boiling hydrochloric acid to reduce the presence of ferromagnetic metals (mainly iron and chromium) down to the ~ 10 ppm level.

#### Structural Characterisation of Sintered Diamond

We also similarly characterised the sample D19 obtained by sintering DP 0–0.05. The D19 XRD pattern was obtained by the same method as that for DP 0–0.05 powder. It was found to display only the five Lorentzian peaks characteristic of a cubic diamond phase (Fig. 3, green line) corresponding to the 111, 220, 311, 400, and 331 planes. There is no evidence of the graphite phase present in the precursor material. The 111 peak dissymmetry is also strongly reduced (Fig. 3, right inset). This suggests a corresponding substantial reduction in stacking faults. Moreover, the *L*_CSR_ coherence length was found to be 10.8 nm, indicating the enlargement of the diamond nanocrystallites during sintering. Considering these observations, we hypothesise that the graphitic and non-cubic diamond phases are converted into the cubic diamond phase during the sintering process. This probably results from the dissolution of these phases, followed by growth of a cubic diamond phase within the interstitial spaces between the crystallites of the polycrystals. Such a process has previously been observed for detonation nanodiamonds [14].

**Fig. 5:**
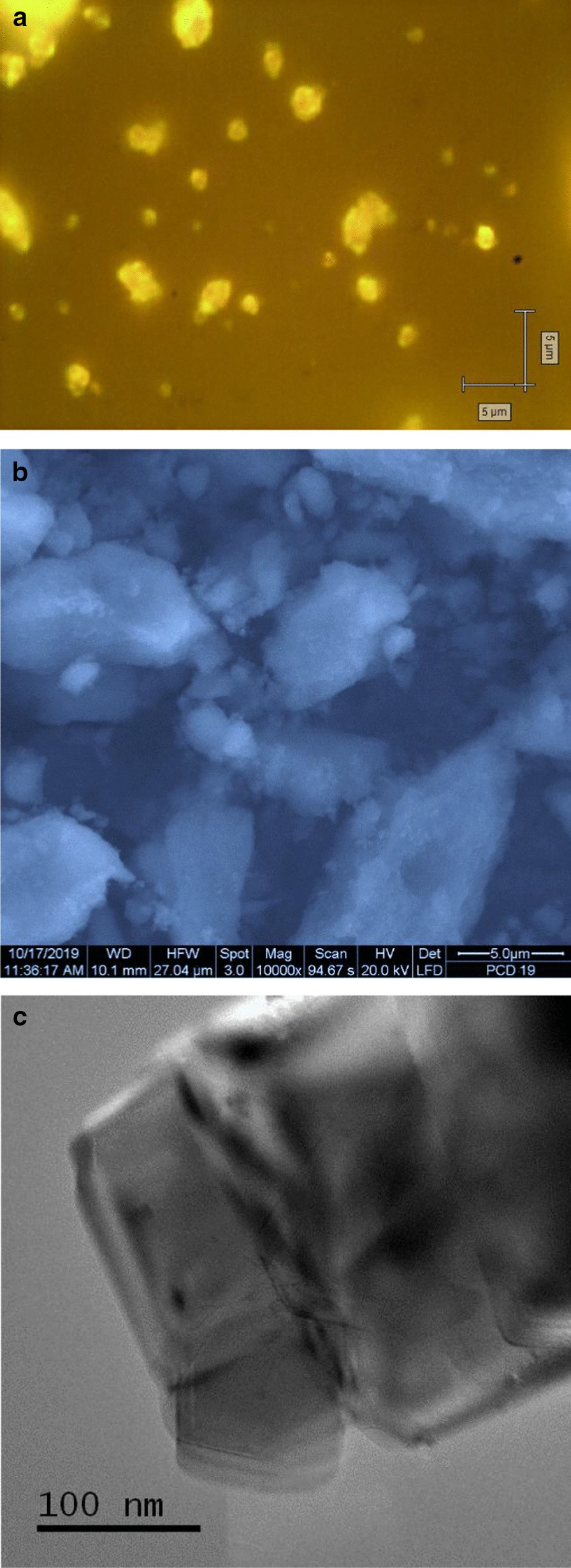
Optical (**a**), scanning electron microscopy (**b**), and transmission electron microscopy (**c**) images of the as-synthesized D19 diamond particles. The optical image was taken with 100× microscopic objective. The corresponding parameters used for taking the electronic SEM image are indicated at the bottom of panel (**b**)

Optical and scanning electron microscopy images of D19 sample are presented in Fig. 5a, b, respectively. Here, we see the particles of arbitrary shape with sizes from ~ 0.3 to ~ 5 microns. They are actually the dense submicron and very loose micron-sized aggregates of much smaller diamond crystallites bound together by covalent bonds or weak van der Waals bonds. Such bonds usually result from the interactions of the surface functional groups of neighbouring diamond particles. The individual diamond particles constituting these loose aggregates can be seen by transmission electron microscopy. A TEM image of a D19 diamond crystallite with size about ~ 100 nm is shown in Fig. 5c. This image was taken using a JEOL JEM-2100F transmission electron microscope (Hosei University) at an acceleration voltage of 200 kV. The sample was fixed on a copper grid without a carbon substrate.

### Methods to Analyse the Content in Defects of Pre- and Post-sintered Nanodiamonds

Both diamond samples were analysed by means of EPR, Raman, and fluorescence spectroscopies. The EPR spectra were recorded at room temperature, at a microwave frequency of 9.444 GHz, using an EPR spectrometer (JES-FA 300, JEOL, Japan). A mass of 20 mg of powder was introduced into a quartz EPR tube of 4 mm in diameter. The height of the powder column in the tube did not exceed 10 mm. The open end of the tube was sealed against moisture.

EPR spectra with *g*-factors in the range *g* = 4.00–4.30 were recorded with microwave power of *P*_MW_ = 10 mW, magnetic field modulation amplitude *A*_m_ = 1 mT and frequency *ν* = 100 kHz, amplifier gain *G* ≈ 10^3^, and *N* = 16 signal accumulation cycles. These parameters were chosen to obtain the optimal signal-to-noise ratio. The time constant was 0.03 s, and the total recording time for the magnetic field sweep over the 130–200 mT interval was 120 s. EPR spectra with *g*-factors *g* ≈ 2 were recorded in the interval from 327 to 347 mT, with microwave power of *P*_MW_ = 0.03 mW, magnetic field modulation amplitude *A*_m_ = 0.035 mT, amplifier gain *G* ≈ 10^2^, and *N* = 4 signal accumulation cycles. Note that, as a rule, for the broad main EPR signals (*g* ≈ 2) with linewidth > 0.5 mT, the peak-to-peak EPR signal intensity (*I*_pp_) follows a MW power dependence *I*_pp_ ~ (*P*_MW_)^1/2^ up to *P*_MW_ ≈ 100 mW. On the other hand, for narrow EPR signals (linewidths < 0.15 mT at low power), *I*_pp_ saturates at *P*_MW_ > 0.05 mW and has a strong shape distortion at higher values (> 4 mW). Such saturation trends were observed for both the polycrystalline precursor and subsequently sintered diamonds.

We acquired photoluminescence (PL) and Raman spectra, with a micro-Raman spectrometer (“inVia”, Renishaw, UK), in conjunction with an optical microscope (Leica, Germany) using a 50× objective (NA = 0.78) and a CCD detector cooled to -70 °C, in a backscattering geometry. The spectra were recorded with a spectral resolution of ~ 2 cm^–1^. We used the two laser lines of an argon-ion laser at 488 nm and 457 nm wavelengths, with intensities less than 20 W cm^−2^ at the focal point on the sample. We recorded spectral images in the StreamLine™ Plus (Renishaw, UK) mode that uses lower excitation laser intensity in the sample plane due to its focus on a 2 × 30 mμ stripe, compared to standard focusing on a micron-sized circle. This strategy limited laser-induced sample damage and local gas phase etching via overheating and oxidation in ambient atmosphere. Further details of this technique and on diamond powder pressing in cylinders of 2 mm diameter have been described previously [19].

Fluorescent images of isolated D19 particles were obtained with confocal wide-field epifluorescence microscopy with a 100× objective. Particles were deposited on a glass coverslip, from the supernatant fraction of a diluted, water-based suspension of D19 powder, via spin coating. The coverslip was preliminarily treated in oxygen plasma to avoid parasitic fluorescence from residual organics and to promote better attachment of the D19 particles to the cover glass. Fluorescence was excited with a 488-nm laser (excitation power ≈ 40 mW) and collected with a 525/40 interference filter. A specially cooled 2D-array CCD detector (camera temperature = − 79.9 °C) was used to record the images. The images (size ~ 80 × 80 μm, 80.00 nm/pixel) were recorded with 60 ms exposure time. A greyscale pallet was used for image presentation. The image was analysed using Fiji software.

## Results

### EPR of Polycrystals and Diamond Crystallites Before and After the Sintering

**Fig. 6:**
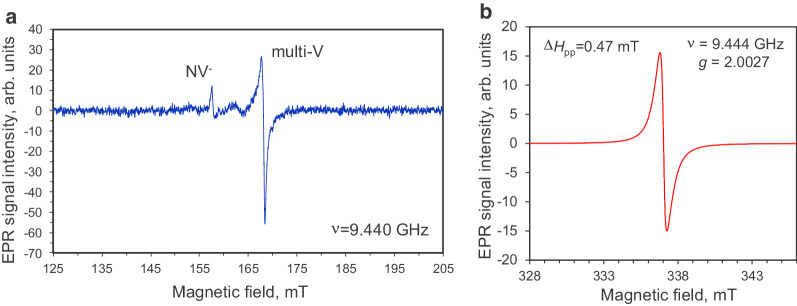
EPR spectra of the DP 0–0.05 fraction of diamond particles in the half magnetic field range (**a**) and around the resonant magnetic field of the singlet with strong signal at *g*-factor *g* ≈ 2.0027 (**b**). Microwave power *P*_MW_: 10 mW (**a**) and 0.03 mW (**b**). Microwave frequency* ν* = 9.44 GHz. Both spectra are registered in the regime far from saturation

The EPR spectra of the DP 0–0.05 polycrystalline diamond powder, in both the half-magnetic field range and the high-field range, are shown in Fig. 6. The EPR spectra registered in these distinct ranges were measured at high and low microwave powers *P*_MW_ = 10 mW and *P*_MW_ = 0.03 mW, respectively. Here the high-field region was normally selected in the vicinity of the absorption line related to the main microwave-induced flip-flop Δ*M*_s_ = 1 transitions of spins *S* = ½, whereas the extended half-field range was specially selected to search for the signals from Δ*M*_s_ = 2 transitions of possible triplet *S* = 1 centres. The half-field EPR spectrum has very low intensity and demonstrates the presence of triplet NV^−^ centres (signal with *g* = 4.27 at *H*_res_ = 158 mT [10, 16]) and triplet multivacancies (signal with *g* = 4.00 at *H*_res_ = 168 mT [16]) in the polycrystalline diamond precursor. Both of these defect types are present in very small concentrations (< 1 ppm); hence, it was necessary to use high microwave power for detection such rare centres. Moreover, in the high-field domain we also observed the characteristic signature of spin-half centres (*g* = 2.0027 at *H*_res_ = 337 mT [10, 16, 21]), but with a broad (Δ*H*_pp_ = 0.47 mT), single Lorentzian-derivative shape without any fine structure [16]. Given the assumption that these spins are from independent centres, this broad, intense signal can be tentatively attributed to C–C dangling bond spins and to exchange coupled paramagnetic nitrogen spins with unresolved hyper-fine structure (HFS).^2^ We estimated the concentration of all paramagnetic species with spin *S* = 1/2 to be ~ 4 × 10^19^ spin/g (800 ppm), which is ~ 1.5 times smaller than the value previously reported for 5 nm detonation nanodiamonds [22].

**Fig. 7:**
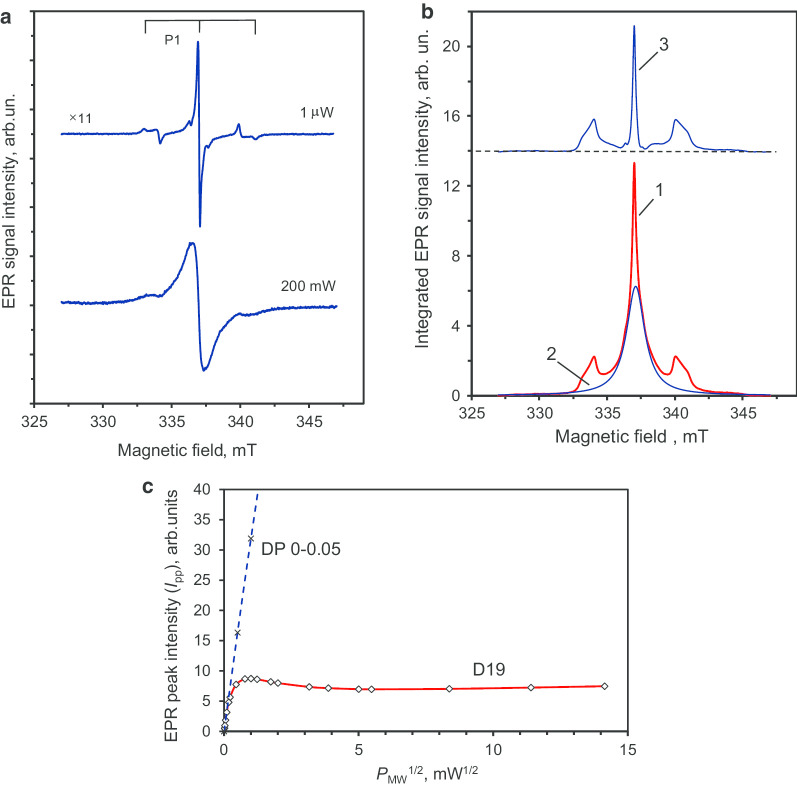
The main first-derivative EPR signal of D19 diamond crystals at low and high microwave powers (**a**), decomposition of the integrated EPR signal into components related to two groups of spins (**b**), and the corresponding saturation trend of the peak intensity of this EPR signal versus the square root of microwave power in the range up to 200 mW (**c**). The triplet HFS structure of the EPR signal of the P1 centre (substitutional nitrogen) is clearly distinguishable in (**a**). In (**b**): curve 3, having triplet structure, corresponds to the EPR spectrum of only P1 centres. In (**c**): the dashed straight line is the *I*_pp_ vs (*P*_MW_)^1/2^ dependence for DP 0–0.05 polycrystals given for reference. Four experimental points at *P*_MW_ = 0.5, 1, 2, 4 mW were used for its plotting (two of them are not presented here). Microwave frequency* ν* = 9.44 GHz

Figure 7a shows the EPR spectra of D19 sample for very low (*P*_MW_ = 1 μW) and very high (200 mW) microwave powers, *i.e.* in the regimes far below saturation or at saturation (as evidenced by broadening), respectively. The spectrum for *P*_MW_ = 1 Wμ has a central line with a narrower linewidth of 0.14 mT compared to that of the precursor particles. Figure 7b (curve 1) displays the doubly integrated low microwave power EPR signal. This signal can be decomposed into: a broad (FWHM ≈ 1.7 mT) Lorentzian-shape signal (Fig. 7b, curve 2) associated with the most abundant *S* = 1/2 species (of high local density and organised into spin clusters) and a very narrow central line with two symmetrical satellite lines separated by ~ 6 mT (Fig. 7b, curve 3). The ratios of the integrated intensities for the two satellite lines relative to the central line are 0.90 and 1.09, respectively. Hence, they have equal integrated intensities within a ± 10% experimental measurement accuracy. The satellite lines are related to the hyper-fine structure of the EPR signal of neutral paramagnetic substitutional nitrogen (^14^ N, *S* = 1/2, *I* = 1), known as P1 centres [23, 24]. P1 centres were not observed in the precursor polycrystalline nanodiamonds but are clearly detected in the diamond after sintering. Previous work has shown that this characteristic triplet HFS structure related to P1 centres appears only in coarse diamond crystals with size exceeding 50–80 nm [25]. Moreover, sintering of detonation nanodiamonds in the presence of ethanol has been demonstrated to promote crystallite enlargement based on diamond recrystallisation and new diamond phase growth [26]. Hence, the current EPR data are in agreement with similar enlargement happening here from individual crystallites of polycrystalline particles with size ~ 7–10 nm to new crystallites of individual size ~ 40–50 nm.^3^

This hypothesis is confirmed not only by the observation of the narrow EPR signal^4^ (*g* = 2.0024) of P1 centres having HFS characteristics [26], but also by the detection of *I*_pp_ saturation above *P*_MW_ = 0.7 mW for the D19 sample. For the precursor polycrystalline nanodiamond DP 0–0.05, the peak intensity of the main EPR signal does not saturate even at a high level of microwave power (*P*_MW_ = 20 mW). The power dependence *I*_pp_ ~ (*P*_MW_)^1/2^ holds well across the whole range of microwave power (*P*_MW_ = 0–20 mW). The *I*_pp_ ~ (*P*_MW_)^1/2^ dependence for DP 0–0.05 sample is shown in Fig. 7c by a dashed line, up to ~ 16 mW microwave power. Such linear dependence with no saturation results from the high concentration of paramagnetic centres within the polycrystalline nanodiamonds and the very short spin–lattice and spin–spin relaxation times. The saturation behaviour of D19 sample *g* = 2.0024 EPR signal is also displayed in Fig. 7c in the 0–200 mW range.^5^ We observe an *I*_pp_ ~ (*P*_MW_)^1/2^ dependence only for microwave power below ~ 15 Wμ. In this case, *I*_pp_ demonstrates saturation in the range below 1 mW and reaches a maximum at ~ 1 mW. The *I*_pp_ then substantially decreases in the 1–25 mW range before slowly increasing again to the maximum power used (200 mW). The presence of such a saturation trend in *I*_pp_ (*P*_MW_), and also the drop above *P*_MW_ = 1 mW, is characteristic of P1 centres with relatively large spin–spin and spin–lattice relaxation times, located far away from other defects and from the particle edges [27]. These conditions are met for P1 centre concentrations smaller than 200 ppm in type Ib HPHT nanodiamonds with size exceeding 60–80 nm. However, the actual mean size of the individual D19 diamond crystallites (elementary particles), as seen from viewpoint of only EPR, is an open question. It can be roughly solved by comparing the actual EPR spectrum of D19 with the series of powder EPR spectra of milled Ib HPHT diamonds with mean size varying from 18 to 390 nm [28]. Following Ref. [28], where these EPR spectra were published, P1 HFS signatures related to substitutional nitrogen are completely absent in powder diamonds with mean size ≤ 30 nm, but still present in the samples of intermediate size (85–130 nm). This comparison indicates that the mean size of synthesized D19 diamond crystallites is in the region of 10,030 ±  nm. This estimation coincides well with the representative size observed in the TEM image shown in Fig. 5c. It is notable that the EPR spectrum of D19 recorded at the high power of *P*_MW_ = 200 mW (Fig. 7a) shows a lack of definition of the HFS structure of the P1 signal. The broad central line suggests the presence, at the nanoscale, of dense clusters of paramagnetic spin-half that strongly couple to each other.

Altogether, the decrease in the linewidth of the main EPR signal (*g* = 2.0024), the appearance of well-defined HFS characteristics in the P1 centre spectrum after sintering, and the *I*_pp_ saturation for *P*_MW_ > 0.7 mW (Fig. 7c) are suggestive of an increase in size by up to one order of magnitude (crystal size > 50 nm). It also indicates a better crystallinity of the nanodiamond in the D19 sample. From van Wyk measurements [29], a smaller amount of paramagnetic defects (< 200 ppm) are expected, based on the narrow linewidth (0.14 mT) of the *g* = 2.0024 main paramagnetic signal in the D19 sample.

### Fluorescence and Raman Scattering of Diamond Crystals

**Fig. 8:**
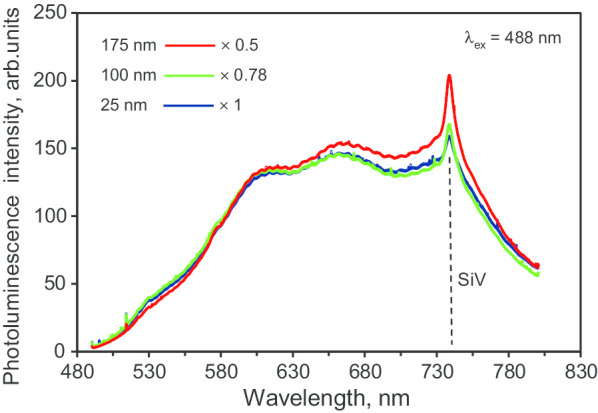
PL spectra of various submicron fractions of Microdiamant™ polycrystalline diamond particles: blue—DP 0–0.05 (mean size 25 nm), green—DP 0–0.2 (mean size 100 nm), red—DP 0–0.35 (mean size 175 nm). Excitation wavelength* λ* = 488 nm. The prominent peak at 738 nm marked by the vertical dashed line is the zero-phonon line of negatively charged SiV^−^ centres, which can be observed in all polycrystalline diamond fractions. For better comparison, the spectra are specially normalized for PL intensity at* λ* = 590 nm. Normalising coefficients are indicated in the figure

The photoluminescence (PL) spectrum of the DP 0–0.05 precursor together with the PL spectra of some much coarser fractions of polycrystalline diamond particles (DP 0–0.2 and DP 0–0.35) produced by Microdiamant^TM^ is shown in Fig. 8. The spectrum of DP 0–0.05 under the 488 nm excitation wavelength has two features of note: the prominent narrow PL line at 738 nm, associated with the zero-phonon line of negatively charged SiV^−^ centres, and a broad spectrum background with PL bands centred at 525, 600, 660 and 740 nm, associated with various light-emitting centres in diamond, including NV centres. For polycrystals with mean size 25 nm (DP 0–0.05), the intensities of these bands are smaller than that for polycrystals with mean sizes of 100 and 175 nm (DP 0–0.2 and DP 0–0.35, respectively). A more detailed analysis of the PL spectra of polycrystalline DP 0–0.05 particles has been previously undertaken [19].

**Fig. 9:**
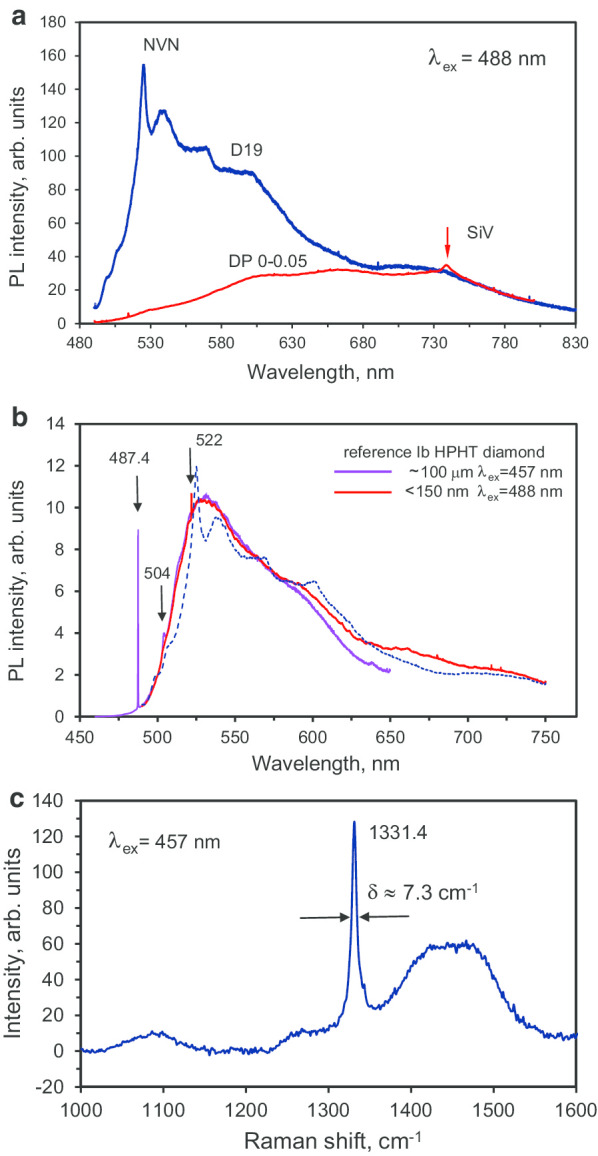
PL spectrum of submicron powder D19 sample at *T* = 293 K (blue line) compared with that of its DP 0–0.05 polycrystalline precursor (red line) under the same conditions with laser excitation at* λ* = 488 nm (**a**), emission spectra, under 457 nm and 488 nm excitation, of two reference synthetic HPHT samples (size ~ 100 μm and < 150 nm) containing NVN centres (**b**) and the Raman spectrum of D19 sample recorded using the 457 nm excitation laser radiation (**c**). Arrows in (**b**): lines at 487.4 nm and 522 nm are single-phonon diamond Raman lines at 457 nm and 488 nm excitation, respectively, and the line at 504 nm is the ZPL of the NVN centres. In (b): dashed line—PL spectrum of D19 at* λ* = 488 nm excitation (for comparison). In (**c**): the diamond Raman line is centred at 1331.4 cm^−1^. *δ* = 7.3 cm^−1^ is a FWHM of diamond Raman line having the Lorentzian shape

Figure 9a shows the PL spectrum of sintered diamond sample D19 (blue curve) at room temperature (RT) together with the PL spectrum of the DP 0–0.05 precursor (red curve). The D19 spectrum displays a green fluorescence characteristic, with a sharp maximum at 525 nm, and a subsequent decrease at larger wavelengths. Note that the single-phonon, sharp Raman line of diamond, which is expected at 522 nm, was too weak to be detected on the ascending slope of the PL signal under 488 nm excitation wavelength.^6^ As previously reported [9, 30], such spectra—with a continuous higher wavelength band of “triangular” shape—are characteristic of optical emission from NVN centres (also known as H3 centres) in submicron (< 140 nm) diamonds at RT. In the PL spectrum of sample D19, at least four broad bands (“bumps”) centred at 538, 569, 601 and 710 nm can be additionally distinguished. We do not believe that they are related to phonon sidebands of the NVN (H3) centres. The origin of the 525 nm sharp peak and “bumps” is still unclear, but it is probably due to an impurity-related complex; the precursor material contains a large number of residual contaminants as mentioned before (see “Structural Characterisation of the Materials Before and After the Sintering” section), some of which are present at significant concentrations (~ 100 ppm). The zero-phonon emission line of NVN at ~ 503 nm wavelength is barely detectable and cannot be distinguished from the two small shoulders (at 500 and 505 nm) in the same region. By comparing the D19 and DP 0–0.05 spectra (Fig. 9a), one can see that they superimpose well for *λ* > 750 nm. However, the spectra differ significantly in the 480–650 nm range due to the appearance after sintering of NVN centres, which were not present in the precursor material. In order to verify our interpretation of the main optical emission of D19 (in the range 500–650 nm) as originating from NVN centres, we compared the PL spectrum of D19 with the PL spectra of two reference samples (HPHT diamonds, Columbus NanoWorks Inc., US) of two very different sizes and both known to contain NVNs (Fig. 9b). The D19 PL spectrum (dashed line) coincides very well with those of the PL spectra of the HPHT microdiamonds containing NVNs. The emission spectrum of the 100-µm sized reference sample shows a sharp single-phonon diamond Raman line (487.4 nm) and the zero-phonon line (504 nm) of NVN under 457 nm laser excitation at room temperature (Fig. 9b, violet spectrum). The 150-nm sized HPHT nanodiamonds were excited at 488 nm, and it displayed a very similar global photoluminescence spectrum shape as that of the 100-µm sized sample, with a Raman single-phonon line at 522 nm. However, it did not exhibit the NVN zero-phonon line, behaving in that sense exactly like the D19 sample. The “bumps” present in the D19 sample spectrum are absent from both reference PL spectra, indicating that these features are not related to the NVN emission.

We also measured Raman scattering from D19, in the range 1000–1600 cm^−1^, under excitation by 457 nm laser radiation. Figure 9c displays the Raman spectrum, corrected to remove the autofluorescence background. The spectrum consists primarily of a narrow characteristic diamond Raman line centred at 1331.4 cm^−1^ and an exceptionally broad (width ≈ 100 cm^−1^) band centred at 1450 cm^−1^. The latter could be due to non-diamond amorphous carbon phase and/or some transpolyacetylene (TPA) species located at diamond crystals surface [1–3]. A further, ill-defined, band at ~ 1090 cm^−1^ of lower intensity is probably related to TPA species. The broad band at ~ 1450–1480 cm^−1^ could also be related to multivacancy chains in the diamond lattice and *sp*^2^- rehybridisation within these chains [6].^7^ Furthermore, we did not observe the characteristic G-band (centred at 1570–1590 cm^−1^) associated with an *sp*^*2*^ graphitic nanophase. These observations are indicative that the diamond sample D19 being graphite free, which is also in agreement with its white colour under daylight illumination.

Moreover, the width of the Raman diamond line (7.3 cm^−1^) in the D19 sample is smaller than that for the DP 0–0.05 polycrystalline particles (10.6 cm^−1^). Table 1 contains Raman diamond line data for bead-milled synthetic Ib HPHT diamonds with mean size varying from 25 to 1000 nm (Microdiamant AG, Switzerland). The linewidth decreases from 9.12 to 5.24 cm^−1^ with increasing size. This can be explained by the lower prevalence of structural defects in larger crystals. Using data as a calibration curve to infer the crystal size from the diamond Raman linewidth of *δ* ≈ 7.3 cm^−1^ yields an estimation for the D19 crystal size of ~ 80 nm. This value coincides reasonably with the estimation in “EPR of Polycrystals and Diamond Crystallites Before and After the Sintering” section on the basis of EPR data. Moreover, this value is very similar to our previous published results where diamonds were obtained by HPHT sintering of 5-nm DND in the presence of ethanol [31]. However, the precursor material used for sintered DND and the one used in this work using the smallest (~ 25 nm) fraction of milled Du Pont shock-wave polycrystalline diamonds are considerably different from the viewpoint of crystal types and elementary crystallite sizes. This obtained size is about 8 times larger than the coherent scattering region length of *L*_CSR_ ≈ 11 nm extracted from XRD earlier, but it is consistent with crystallite having a low density of structural defects, in agreement with the EPR studies.

**Table 1:** The FWHM of the diamond Raman line (at 1331.4 cm^−1^) for some selected fractions of bead-milled synthetic monocrystalline Ib HPHT diamonds with variable median size

Mean size of selected diamond powder fraction, nm	25	75	100	180	≥ 1000
Raman linewidth^a^, cm^−1^	9.12	7.98	7.23	7.05	5.24

^a^The linewidths are not corrected from the Raman spectrometer resolution of about 2 cm^−1^

**Fig. 10:**
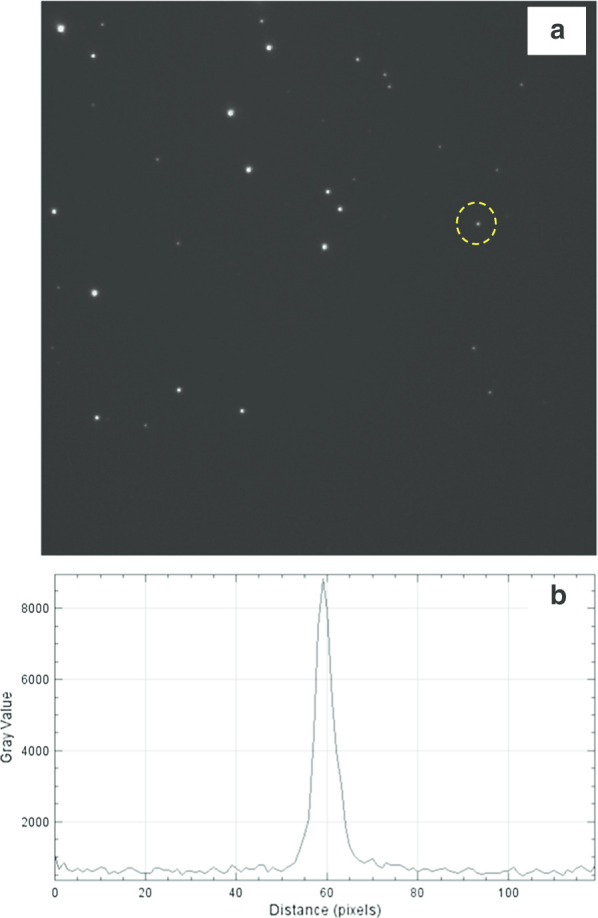
Wide-field fluorescent image of isolated D19 particles obtained by confocal epifluorescence microscopy (**a**), and the intensity profile of the selected nanoparticle marked by the yellow circle (**b**). Image size: ~ 80 × 80 mμ. One pixel corresponds to 80 nm

We also studied the fluorescence from very fine individual D19 particles. For this purpose, the supernatant fraction of diluted and ultrasonicated water suspension of D19 particles obtained after centrifugation at 4500 × g for a 30 min was used. Coarse particles and large loose aggregates with size exceeding ~ 0.2–0.3 micron were absent in such supernatant. Fine D19 particles were spin-coated onto a thin glass coverslip from the diluted supernatant of D19 particles. A typical image of fine fluorescent D19 particles is shown in Fig. 10a in greyscale (mono 14-bit images). It consists of many spots with different brightness. Some spots, such as that marked by the yellow circle, have sizes close to the diffraction limit. The intensity profile of this spot has a Gaussian shape in its central core and a FWHM of about 5–6 pixel corresponding to ~ 440 nm (Fig. 10b). Such spots come from at least quarter-micron particles and particles of smaller size. A greater number of brighter spots correspond to the larger reassembled aggregates of D19 particles having more NVN colour centres and hence the overall emission intensity increasing.

## Discussion

We showed that under HPHT conditions, and in the presence of ethanol, we can convert polycrystalline diamond particles (composed of tightly cemented nanometre-sized cubic diamond crystallites separated by a non-cubic diamond phase) into larger cubic diamond crystallites. The process probably occurs through recrystallisation of the cubic diamond phase and transformation of non-cubic diamond phases including multiple twin boundaries into diamond. During this process, vacancies appear and can form NVN complexes with nitrogen atom pairs. These complexes have a characteristic photoluminescence in the green. While the EPR spectra of the precursor polycrystalline diamonds show NV^−^ triplet centres, triplet multivacancies, and SiV^−^ centres, none of these were present after the sintering. The disappearance of NV^−^ centres and multivacancies has previously been observed [31] after sintering detonation nanodiamonds at HPHT conditions (*P* = 7 GPa and *T *≥ 1350 °C) with ethanol.^8^ The presence of multivacancies is a characteristic feature of damaged diamond lattices, with defects mainly located in a thin layer of ~ 2 nm at the surface. The absence of the paramagnetic triplet and SiV colour centres is strong evidence that substantial recrystallisation took place, accompanied with the appearance of new defect types (NVN). The saturation trend of the substitutional nitrogen (P1 centre) EPR signal with increasing microwave power indicates long spin–spin and spin–lattice relaxation times. These are signatures of improvement of the quality of diamond crystal lattice after sintering. We can assume that during HPHT sintering, vacancies from empty spaces within or between polycrystals join with A-centres to form the new NVN entities.

## Conclusions

Sintering of diamond polycrystals, with size varying from 3 to 50 nm, in the presence of ethanol, lead to the substantial enlargement of elementary diamond nanocrystals and improved their crystalline quality. During this process, SiV^−^ and NV^−^ colour centres present in the precursor nanodiamond disappeared, while the EPR signature of P1 substitutional nitrogen paramagnetic centres appeared. We also observed the green photoluminescence of NVN colour centres. The comparison of the FWHM of diamond Raman line (~ 1332 cm^−1^) of the synthesised selected microcrystals under study with those of a series of reference samples revealed that the mean size of diamond crystals after sintering is approximately 80 nm. The analysis of the EPR spectrum dependence upon microwave power demonstrated the good crystalline quality of the synthesised sintered diamond with a concentration of P1 centres smaller than 200 ppm. Hence, our technique of HPHT sintering is a strong alternative to conventional high-energy particle beam irradiation [9] to form NVN centres in nanodiamond. It can be used to produce purely “green” fluorescing nanodiamonds with no (or very limited) crosstalk with the “red” fluorescing nanodiamonds (containing NV^0^ and NV^−^ centres), as required in biolabelling for cathodoluminescence integrated correlation electron-light microscopy [32].

## Data Availability

The data underpinning this manuscript is available from the corresponding author on request.

## Abbreviations

NV^−^: Nitrogen vacancy
NVN: Nitrogen-vacancy-nitrogen
HPHT: High-pressure, high-temperature
DND: Detonation nanodiamond
EPR: Electron paramagnetic resonance
FWHM: Full width at half maximum
XRD: X-ray diffraction
L_CSR_: Coherent scattering region length
HRTEM: High-resolution transmission electron microscopy
PL: Photoluminescence
MW: Microwave
HFS: Hyper-fine structure
RT: Room temperature

## Footnotes

1 The apparatus response function of the XRD diffractometer was determined from a LaB_6_ reference sample.

2 One mechanism explaining the lack of HFS in the EPR signal of paramagnetic nitrogen in size < 50 nm diamond nanoparticles is described in detail elsewhere [21]. Alternative mechanisms are also not excluded [33, 34].

3 X-ray diffraction CSR size is ~ 4 times smaller in this case.

4 This narrow EPR signal is slightly asymmetrical and consists of at least two components related to P1 centres and to defects having C–C dangling bond spins *S* = 1/2. The intensity of the second component represents at least 30% of the intensity of the main peak.

5 Here we assume the sequence of untreated first-derivative experimental EPR spectra measured at different *P*_MW_, two of which are shown in Fig. 7a.

6 However, the Raman line was detected at 457 nm excitation.

7 The Raman band centred at ~ 1480 cm^−1^ was found for HPHT diamonds grown with magnesium-based catalysts [6]. Such diamond crystals may contain SiV and GeV colour centres.

8 Instead of a multivacancy *g* = 4.00 singlet EPR signal, a new EPR signal with a quintet hyperfine structure related to N⋯N pairs separated by no more than 0.7 nm was observed in this case.
